# CNV analysis in the Lithuanian population

**DOI:** 10.1186/s12863-016-0373-6

**Published:** 2016-05-04

**Authors:** A. Urnikyte, I. Domarkiene, S. Stoma, L. Ambrozaityte, I. Uktveryte, R. Meskiene, V. Kasiulevičius, N. Burokiene, V. Kučinskas

**Affiliations:** Department of Human and Medical Genetics, Faculty of Medicine, Vilnius University, Santariskiu St. 2, LT-08661 Vilnius, Lithuania; Clinics of Internal Diseases, Family Medicine and Oncology, Faculty of Medicine, Vilnius University, Santariskiu St. 2, LT-08661 Vilnius, Lithuania; Master of Science (MSc), Bioinformatics student, VU University Amsterdam, Amsterdam, Netherlands

**Keywords:** CNV, CNVRs, Copy number variation, LITGEN project

## Abstract

**Background:**

Although copy number variation (CNV) has received much attention, knowledge about the characteristics of CNVs such as occurrence rate and distribution in the genome between populations and within the same population is still insufficient. In this study, Illumina 770 K HumanOmniExpress-12 v1.0 (and v1.1) arrays were used to examine the diversity and distribution of CNVs in 286 unrelated individuals from the two main ethnolinguistic groups of the Lithuanian population (Aukštaičiai and Žemaičiai) (see Additional file 3). For primary data analysis, the Illumina GenomeStudio™ Genotyping Module v1.9 and two algorithms, cnvPartition 3.2.0 and QuantiSNP 2.0, were used to identify high-confidence CNVs.

**Results:**

A total of 478 autosomal CNVs were detected by both algorithms, and those were clustered in 87 copy number variation regions (CNVRs), spanning ~12.5 Mb of the genome (see Table 1). At least 8.6 % of the CNVRs were unique and had not been reported in the Database of Genomic Variants. Most CNVRs (57.5 %) were rare, with a frequency of <1 %, whereas common CNVRs with at least 5 % frequency made up only 1.1 % of all CNVRs identified. About 49 % of non-singleton CNVRs were shared between Aukštaičiai and Žemaičiai, and the remaining CNVRs were specific to each group. Many of the CNVs detected (66 %) overlapped with known UCSC gene regions.

**Conclusions:**

The ethnolinguistic groups of the Lithuanian population could not be differentiated based on CNV profiles, which may reflect their geographical proximity and suggest the homogeneity of the Lithuanian population. In addition, putative novel CNVs unique to the Lithuanian population were identified. The results of our study enhance the CNV map of the Lithuanian population.

**Electronic supplementary material:**

The online version of this article (doi:10.1186/s12863-016-0373-6) contains supplementary material, which is available to authorized users.

## Background

Human genome variation embodies single nucleotide polymorphisms (SNPs), copy number variants (CNVs), small deletions and insertions (INDELS), and large chromosomal aberrations (size >2–5 Mb) [1]. Until the discovery of copy number variation (CNV), SNPs were thought to be the predominant form of genetic and phenotypic human variation. Today it is known that CNV plays a significant role in genomic heterogeneity [2]. Copy number variants are defined as DNA segments ranging from 1 kb to several Mb and are present in variable copy number compared with a reference genome [3, 4]. These segments can be deleted, duplicated, inserted, inverted or translocated. CNVs can span from 4.8 to 9.5 % of the autosomal genome, suggesting that significant portions of the genome have the potential to vary in copy number within the normal population [5]. According to McCarroll SA et al. [6] approximately 80 % of the observed copy number differences of DNA segments between pairs of individuals are common copy number polymorphisms (CNPs) with an allele frequency of >5 %, and more than 99 % of them are inherited. The current 1000 Genomes phase 3 study indicates that the bulk of structural variations (SV) occur at low frequency (65 % exhibit a variant allele frequency (VAF) of <0.2 %) and are consistent amongst individual SV classes [7]. Several studies have associated CNVs with complex human diseases, such as selected autoimmune diseases, HIV, tumours, psychiatric disorders, intellectual disability, schizophrenia, and autism [8–13].

Several technological approaches such as array comparative genomic hybridisation (aCGH), SNP array technologies, and next generation sequencing are used to detect CNV [14–16]. Numerous CNV prediction algorithms have been developed for CNV calling [17–20].

Although there have been studies that analysed some CNV properties in HapMap samples and large population cohorts, the knowledge of the characteristics of CNVs between unique populations and within the same population is incomplete [3, 8, 21–24]. Moreover, the CNV results from different studies are limited due to the difficulties of data consolidation. Furthermore, different ethnic groups (unique populations) represent differences in genomic CNV distribution that may contribute to phenotypic variation and differences in susceptibility to diseases [3, 22, 25]. A catalogue of reference CNVs derived from patients and the general population can help to make an accurate clinical interpretation of CNVs detected in patients. Existing CNV databases do not contain a full spectrum of data about specific populations.

The aim of this study was to perform a comparative evaluation of CNV characteristics in the Lithuanian population to address questions about the origin and genetic structure of the present day population. The main interest was to elucidate genetic differences between the two main ethnolinguistic groups (Aukštaičiai and Žemaičiai) of Lithuania, since historically the Aukštaičiai and Žemaičiai probably developed over a long period of time as two independent Baltic tribes [26].

Illumina 770 K HumanOmniExpress-12 v1.0 and HumanOmniExpress-12 v1.1 arrays were used to investigate CNVs in 286 unrelated individuals from the Lithuanian population. CNV analysis was carried out using cnvPartition 3.2.0 (Illumina Inc., USA) and QuantiSNP 2.0 calling algorithms [17]. Two different algorithms were afterwards used to identify the high-confidence CNVs clustered in the copy number variable regions (CNVRs). Furthermore, comparative CNV and CNVR analysis between the two main ethnolinguistic groups in the Lithuanian population (Aukštaičiai and Žemaičiai) was performed.

The results not only complement current knowledge of structural variation but also are fundamental for future genomic studies of the Lithuanian population.

## Results

### CNV characteristics

Aiming to discover the genetic differences between two main ethnolinguistic groups in the Lithuanian population (Aukštaičiai and Žemaičiai), we analysed a total of 286 samples (*n* = 166 for Aukštaičiai and *n* = 120 for Žemaičiai).

A summary of the characteristics of the CNVs and CNVRs identified in the Lithuanian population and ethnolinguistic groups (Aukštaičiai and Žemaičiai) is shown in Table 1.

**Table 1:** Characteristics of CNVs and CNVRs in the Lithuanian population

CNVs	Aukštaičiai	Žemaičiai	Overall
Sample size	166	120	286
CNV carriers	103 (62 %)	84 (70 %)	187 (65.4 %)
Number of CNVs identified	262	216	478
CNVs per person	1.58	1.8	1.67
Duplications	123 (47 %)	103 (47.7 %)	226 (47.3 %)
Deletions	139 (53 %)	113 (52.3 %)	252 (52.7 %)
Mean size of CNVs identified	133 kb	152.8 kb	141.9 kb
Median size of CNVs identified	70.7 kb	86.2 kb	78.2 kb
CNVRs			
Total number of CNVRs identified	49	38	87
Mean size of CNVRs identified	138.4 kb	144.1 kb	143.7 kb
Median size of CNVRs identified	73.6 kb	85 kb	86.8 kb
Genome coverage by CNVRs	6.8 Mb	5.5 Mb	12.5 Mb

After the combined analysis of CNV calling by two algorithms (QuantiSNP 2.0 and cnvPartition 3.2.0), there were 478 autosomal high-confidence CNVs identified in 65.4 % of the individuals analysed. The length of the CNVs ranged from 4.9 kb to 1.38 Mb, with a mean size of 141.9 kb and a median size of 78.2 kb (Table 1). More than half the CNVs identified (~52 %) were small in size and were 50–200 kb. The average number of CNVs per person was 1.67, the number of CNVs ranged from 1 to 10 per person. Furthermore, deletions were slightly more abundant (52.7 %) than duplications (47.3 %).

**Fig. 1:**
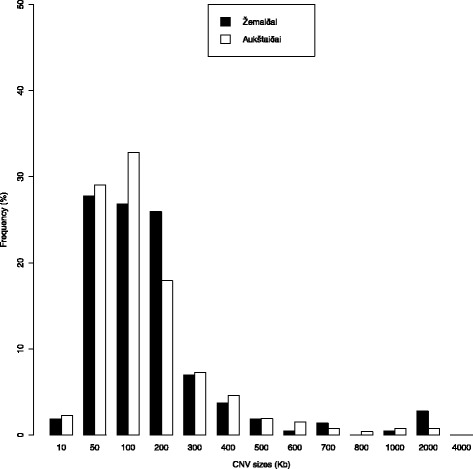
Size distribution of CNVs in the Lithuanian population

**Fig. 2:**
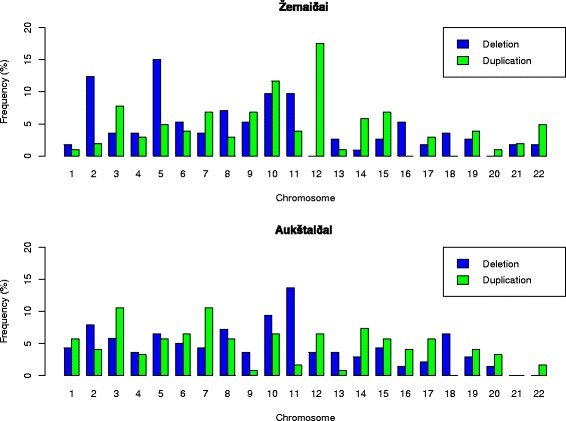
Genomic distribution of CNV deletions and duplications in ethnolinguistic groups of the Lithuanian population

The numbers of CNVs identified were different in the Lithuanian ethnolinguistic groups: 262 CNVs were identified in the Aukštaičiai group (mean size of CNV was 133 kb and the median size was 70.7 kb) versus 216 in the Žemaičiai group (mean size of CNV was 152.8 kb and median size was 86.2 kb). CNVs identified in the Žemaičiai group were larger in size compared with the Aukštaičiai group (Fig. 1). No statistically significant difference in CNV sizes between groups was found (*p* value 0.4206; α = 0.05, Wilcoxon-Mann–Whitney-Test). The percentage of duplications and deletions identified in the ethnolinguistic groups was similar (~47 % duplications, ~53 % deletions) (Table 1 and Fig. 2). The average number of CNVs identified per individual was different in the ethnolinguistic groups. The Aukštaičiai group had 1.58 CNVs per person while the Žemaičiai group had 1.8. No CNVs were identified in approximately 40 % of individuals from the Aukštaičiai group and 30 % from the Žemaičiai group.

### Characteristics of CNVRs

A total of 87 autosomal CNVRs, covering ~12.5 Mb of the autosomal genome, were clustered. CNVRs were identified across all autosomes except the 13^th^, 20^th^ and 21^st^. The genomic distribution of the CNVRs identified in the Lithuanian population is shown in Additional file 1. The size of the CNVRs ranged from 10.6 kb to 1.38 Mb, with a mean size of 143.7 kb and median size of 86.8 kb (Table 1). There were more CNVRs with duplications than those with deletions (50.7 % versus 49.2 %). The mean size of the CNVR duplications was larger (171.4 kb) than the mean size of the CNVR deletions (130.3 kb). Only 13 CNVRs contained both types of variants (deletions and duplications).

There were 49 autosomal CNVRs identified in the Aukštaičiai group. CNVRs (ranging in size from 10.6 to 947.7 kb) covered 6.8 Mb of the autosomal genome and had a mean size of 138.4 kb and median size of 73.6 kb. In the Žemaičiai group, there were 38 clustered CNVRs, which (ranging in size from 7.9 kb to 1.3 Mb) covered 5.5 Mb of the autosomal genome with a mean size of 144.1 kb and median size of 85 kb (Table 1). The difference in CNVR size between ethnolinguistic groups was statistically insignificant (*p* value 0.7976; α = 0.05, Wilcoxon-Mann–Whitney-Test). According to the distribution of CNVRs across autosomes in both ethnolinguistic groups, CNVRs were distributed as in the Lithuanian population: across all autosomes except the 13^th^, 20^th^ and 21^st^.

**Fig. 3:**
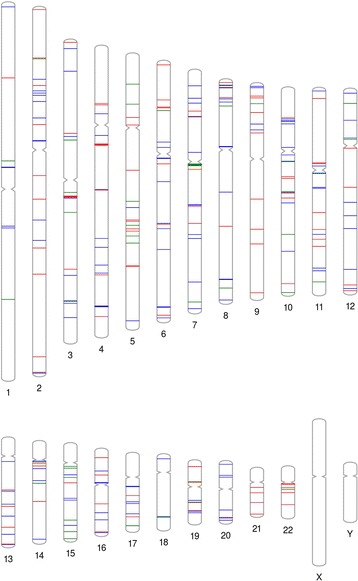
Genomic map of shared and unique CNVRs of the Aukštaičiai and Žemaičiai. Singleton CNVs included. Blue represents unique CNVRs of the Aukštaičiai, red is the unique CNVRs of the Žemaičiai, and green is shared CNVRs. PhenoGram was used to plot and visualise shared and unique CNVRs on the chromosomes [44]

CNVRs were used to analyse CNV sharing between the ethnolinguistic groups. We defined shared CNVR that contained totally or partially overlapped CNVs from both Lithuanian ethnolinguistic groups. We considered only non-singleton CNVRs identified within the Lithuanian population. In total, ~49 % of the non-singleton CNVRs were shared by the Aukštaičiai and Žemaičiai, and the remaining CNVRs were specific to each of the ethnolinguistic groups (Fig. 3). Only one of the shared CNVRs was novel, which overlaps a pseudogene.

### Frequency analysis of CNVs and CNVRs

CNVs were classified as non-singleton if detected in more than one individual and as singleton if detected in only one individual. There were a total of 196 (41 %) singleton and 282 (59 %) non-singleton CNVs identified, and thus non-singleton CNVs dominated. In addition, there were 127 (48.5 %) singleton CNVs in the Aukštaičiai group and 109 (50.5 %) in the Žemaičiai group.

More than a half (57.5 %) of the CNVRs identified in the study population were rare (frequency <1 %). Common CNVRs (frequency ≥5 %) comprised 1.1 % of all CNVRs identified. The majority of CNVR frequencies in the ethnolinguistic groups ranged from 1–5 %, whereas 2 % of all CNVR frequencies in the Aukštaičiai group and 8 % in the Žemaičiai group ranged from 5–10 %.

No statistically significant differences in the frequencies of CNVs and CNVRs were identified between the ethnolinguistic groups (*p* value 0.9585; α = 0.05, Kendall’s Tau-b rank correlation coefficient test).

### Novel CNVs

After a comparison of the study results with previously reported CNVs in the Database of Genomic Variants (DGV, latest updated: October, 2014), it was found that 91.4 % of the identified CNVs overlapped with CNVs in the DGV and the remaining 8.6 % were not found in the DGV [27]. All novel CNVs were rare, with a frequency of 0.3 %.

In the Aukštaičiai group 92.4 % of the CNVs identified overlapped with those in the DGV and 7.6 % were novel, whereas in the Žemaičiai group 90.3 % of CNVs identified overlapped with published CNVs and the remaining 9.7 % were novel.

### CNV annotation

For biological interpretation of CNV data, the Scripps Genome Annotation and Distributed Variant Interpretation Server (SG-ADVISER) was employed [28]. A great majority of CNVs (~66 %) overlapped known UCSC (http://genome.ucsc.edu/) gene regions. Duplications appeared to overlap known genes more frequently than the deletions (35 % versus 30 %). Based on the annotation results, there were CNV variants as possibly causal to both Mendelian and complex diseases. The most common diseases were cancer, diabetes mellitus, autism, and Prader-Willi syndrome. Between the functional categories of genes that were most enriched within CNVs were cell adhesion, ion transportation, regulation of transcription, sensory perception of smell, and cell signalling. Detailed information about annotated CNVs can be found in Additional file 2.

## Discussion

CNVs are major contributors to genomic structural variations spanning from 4.8 to 9.5 % of the autosomal genome and varying within and among different populations [5, 23]. Analysis of CNV in the Lithuanian population has only recently begun and the results are primary and provisional [29, 30].

Historically, the Aukštaičiai and Žemaičiai developed over a long period of time as two independent Baltic tribes [26]. During the mediaeval period, the Baltic tribes became strongly intermingled and anthropological differences practically disappeared. The Lithuanian population is homogeneous in the context of Eastern Europe or the whole of Europe. Since the Neolithic period, the native inhabitants of the territory of Lithuania have not been replaced by any other ethnic group. In other words, the roots of the present-day Lithuanian population are deep, and the probability that the inhabitants of present-day Lithuania have preserved the ancient genetic composition is high [31]. Previous studies showed minor differences between Aukštaičiai and Žemaičiai in blood groups (P, LW) and genetic markers (TPA25), which might reflect differences in their original gene pools [32]. However, according to Kasperavičiūtė D. et al. [33], results concerning mtDNA HV1 sequence and RFLP polymorphism, and Y chromosomal biallelic and STR variation, Lithuanians are a genetically homogeneous population.

The main interest of this study was to elucidate genetic differences based on CNV profiles between the two main ethnolinguistic (Aukštaičiai and Žemaičiai) groups in Lithuania, as well as in the general Lithuanian population.

CNVs were determined by using SNP microarray genotyping data and two different CNV calling algorithms.

We found that the majority of individuals in the Lithuanian population (65.4 %) carried at least one CNV in common with the CNVs in published studies [8]. The median size of the CNVs identified (78.2 kb) corresponds with the one reported by Redon et al. (81 kb by the 500 K EA platform) [3]. Other CNV characteristics are consistent with the results of other authors [8].

Intrapopulation CNV analysis showed different profiles of CNVs between the ethnolinguistic groups. There were differences in CNV size distribution and CNV incidence between the Žemaičiai and Aukštaičiai groups. More than 60 % of the CNVs detected were specific to each ethnolinguistic group and ~40 % were shared. Analysis of non-singleton CNVR sharing revealed that both Lithuanian ethnolinguistic groups share ~49 % of CNVRs. For example, an analysis of CNV sharing conducted by Haiyi Lou et al. [34] showed that up to 80 % of all non-singleton CNVRs were shared by at least two Chinese ethnic groups, while populations from different continents shared ∼ 40 % of CNVs, and populations on the same continent shared ∼ 50 %.

The moderate CNVR sharing between the Lithuanian ethnolinguistic groups could be explained either by sampling variances, recent evolutionary events, or deleterious effects. In accordance with previous studies, our results support that only common CNVs, which are likely to be of more ancient origin, appear to be shared among populations regardless of ethnicity [35]. Besides, it is known that the distribution of CNVs within and between populations is shaped by mutation, selection and demographic history [3].

Analysis of CNV diversity revealed that novel CNVs were more abundant in the Žemaičiai group (9.7 %) than in the Aukštaičiai group (7.6 %). In the Žemaičiai group, 15.4 % of novel CNVs had a frequency of more than 1 %, whereas in the Aukštaičiai group 20 % of novel CNVs had a frequency of >1 %, and the remaining were singletons in both groups. Among novel CNVs, there were three non-singleton CNV regions. Considering the ethnolinguistic groups separately, we detected two novel non-singleton CNV duplicated regions in the Žemaičiai group. One such region (61.33 kb) was found in chr3:113598223–113659549 overlapping protein coding gene *GRAMD1C* and the other was found in chr4: 179159462–179391633, the intergenic region. Although it is known that *GRAMD1C* functions as binding protein and is an integral component of the membrane (NCBI Gene database information), no phenotype has yet been associated with the *GRAMD1C* gene. Only one novel non-singleton deleted region in chr3: 172740452–172786148 (45.69 kb) was identified in the Aukštaičiai group. This region overlaps with the protein-coding gene *SPATA16*, which is associated with spermatogenesis and male infertility. Considering novel singleton CNVs, we observed that in the Žemaičiai group there were more CNV variants possibly causal to disease than in the Aukštaičiai group. The genes containing CNVs may play a role in disease in Lithuania, but the effect of the variants needs to be confirmed.

Most of the individual CNVs (57.5 %) were found to be rare (<1 %) in the studied population. The reason for the low frequency of CNVs might be the possibility of deleterious effects or the recent occurrence of these variants [36, 37]. McCarroll et al. reported that many CNVs observed at ∼ 1 % within a population share a single mutational origin [6]. These observations suggest that many of the higher frequency CNVs are not due to a substantially higher mutation rate but rather due to a relaxed purifying selection compared with rare CNVs [38].

Corresponding with other reported studies [39, 40], a relatively high number of CNVs that overlap genes were discovered. This could be explained by a high GC nucleotide content in gene-rich regions that are subject to copy number change events [37]. Moreover, the predominant type of CNV that overlapped with regions of genes was duplications. This finding coincides with the hypothesis that duplications are less phenotypically harmful than deletions, and the evolutionary sense of that might be illustrated by Conrad et al. [41], who stated that CNV deletions are relatively gene-poor, implying that many gene-containing deletions are subject to purifying selection.

In this study, analysis of the CNV profile of ethnolinguistic groups in the Lithuanian population did not reveal statistically significant differences between the groups, which may reflect their geographical proximity and suggest the homogeneity of the Lithuanian population. To strengthen current findings, a higher sample size is needed to specify the frequencies of singleton CNVs.

The results of our study not only provide a more comprehensive map of CNVs in the Lithuanian genome but also unravel the main characteristics of CNVs and enrich current scientific knowledge about genomic CNV data of the small, unique European population of Lithuania.

## Conclusions

In summary, we identified 478 high-confidence CNVs in 286 unrelated individuals of the Lithuanian population by two different CNV calling programs based on SNP genotyping data. Afterward, 87 CNVRs were clustered, spanning approximately 12.5 Mb of the autosomal genome. Most individual CNVs were found to be rare (<1 %) in the population studied. In addition, putative novel CNVs unique to the Lithuanian population were identified. The ethnolinguistic groups of the Lithuanian population could not be differentiated based on CNV profiles, however the results of our study enhance the CNV map of the Lithuanian population.

## Methods

### Sample

Lithuania could be divided into six ethnolinguistic groups: three groups of Aukštaičiai (western, southern and eastern) and three groups of Žemaičiai (northern, western and southern) (see Additional file 3). There were a total of 286 study participants randomly selected from the two main ethnolinguistic groups: Aukštaičiai (166 individuals) and Žemaičiai (120 individuals).

This study is part of the LITGEN project, which was approved by the Vilnius Regional Research Ethics Committee No. 158200–05–329–79, date: 2011–05–03. Written informed consent was received from all of the study participants.

### Genotyping

Genomic DNA was extracted from whole venous blood using either the phenol-chloroform extraction method or the automated DNA extraction platform TECAN Freedom EVO (TECAN Group Ltd., Männedorf, Switzerland) based on the paramagnetic particle method. DNA concentration and quality were measured by a NanoDropR ND-1000 spectrophotometer (NanoDrop Technologies Inc., USA).

SNP genotyping of 254 samples was performed with Illumina HumanOmniExpress-12 v1.1 arrays at the Department of Human and Medical Genetics, Faculty of Medicine, Vilnius University, Lithuania, while the other 32 samples underwent this procedure with a HumanOmniExpress-12 v1.0 (Illumina, San Diego, CA, USA) at the University of Tartu, Estonia using the standard Illumina Infinium® HD Assay Ultra protocol recommended by the manufacturer (Catalog # WG-901–4005).

Genotyping data quality control was performed according to the standard recommendations by the manufacturer. Individuals with a call rate <98 % and a standard deviation (SD) of the log R ratio (LRR) of >0.3 were excluded from further analysis.

### CNV calling and CNVR determination

The log R ratio and B allele frequency (BAF) were exported from the normalised Illumina data through the GenomeStudio v2011.1 program to perform CNV calling. Two algorithms available for Illumina data were applied: cnvPartition 3.2.0 (Illumina, San Diego, CA, USA) and QuantiSNP 2.0 [16]. We employed two CNV calling algorithms because CNV calls from two or more algorithms are more reliable with strict filtering (seven or more consecutive SNPs) and reduce the false positive rate in comparison with a single algorithm [35]. After CNV detection by QuantiSNP 2.0, to minimise false positive results we included CNVs for further analysis with at least seven consecutive SNPs (number of probes) and a maximum log Bayes factor (MaxLogBF) of ≥30. Moreover, CNV quality measures SD of LRR (St.Dev.LRR) 0.1–0.25 and SD of BAF (St.Dev.BAF) <0.04 were applied. CnvPartition 3.2.0 was run with default settings, including a confidence threshold of 35 and a minimum homozygous region size of 1,000,000, and the minimum probe count was increased from 3 to 7. CNV calls were accepted if the CNVs were identified by both algorithms at the same locus with at least 50 % overlapping length and the type of copy number change was consistent. CNVs detected on the X chromosome were excluded from further analysis due to the high false-positive rate.

CNVR was defined as a region of overlapping CNVs according to Redon et al. [3]. Thus we constructed each CNVR by taking each CNV identified and expanding its region if an overlap of at least one base position with another CNV occurred. We used a script (provided in Additional file 4) that was developed in house and written in the Python programming language [42]. To determine whether the CNVs identified and CNVRs constructed were novel variants, they were compared to those in the Database of Genomic Variants.

### Statistical analysis

For data manipulation and statistical analysis, R package version 3.0.2. was used [43]. The data were not normally distributed. The evaluation of the CNV and CNVR size difference between the ethnolinguistic groups was performed with the use of the non-parametric Mann–Whitney-Wilcoxon test, significant threshold set to 0.05. Correlation analysis of CNV and CNVR frequencies in both groups was performed by the Kendall’s Tau-b correlation coefficient with α = 0.05.

### Ethics approval and consent to participate

This study was approved by the Vilnius Regional Research Ethics Committee No. 158200–05–329–79, date: 2011–05–03. Written informed consent was received from all of the study participants.

### Consent for publication

Not applicable.

## Availability of data and material

The raw SNP data supporting the conclusions of this article will not be shared due to participant privacy.

## Additional files

Additional file 1: Genomic distribution of CNVRs identified in the Lithuanian population. CNVRs were identified across all of the autosomes except the 13^th^, 20^th^ and 21^st^ (which are not included in the graphic). (PDF 6 kb) Additional file 2: Summary of CNVs detected. Also includes CNV annotation results. (XLSX 368 kb) Additional file 3: Map of ethnolinguistic groups in the Lithuanian population. Six ethnolinguistic groups are distinguished in Lithuania: three groups of Aukštaičiai (East, South, West) and three groups of Žemaičiai (North, South, West). (PDF 317 kb) Additional file 4: Programme for CNVR formation. Includes README_16_02_2014.txt file. (PY 12 kb)
