# Supplementary figures and images for: CNV analysis in the Lithuanian population
Source: BMC Genet. 2016 May 4;17:64. doi: 10.1186/s12863-016-0373-6 (PMC4855864; doi:10.1186/s12863-016-0373-6)

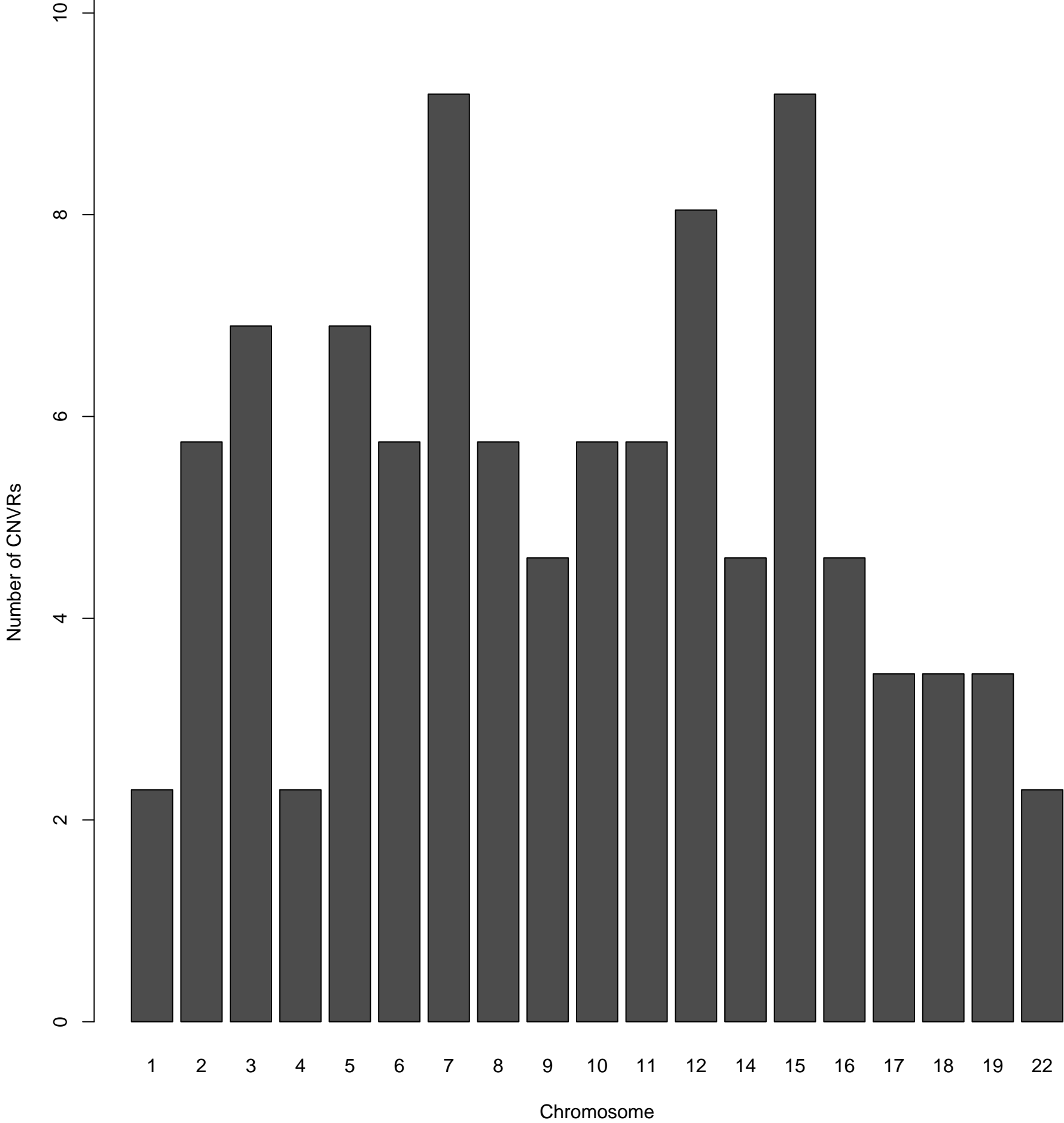

Supplement: Additional file 1: — Genomic distribution of CNVRs identified in the Lithuanian population. CNVRs were identified across all of the autosomes except the 13th, 20th and 21st (which are not included in the graphic). (PDF 6 kb) [file 12863_2016_373_MOESM1_ESM.pdf]

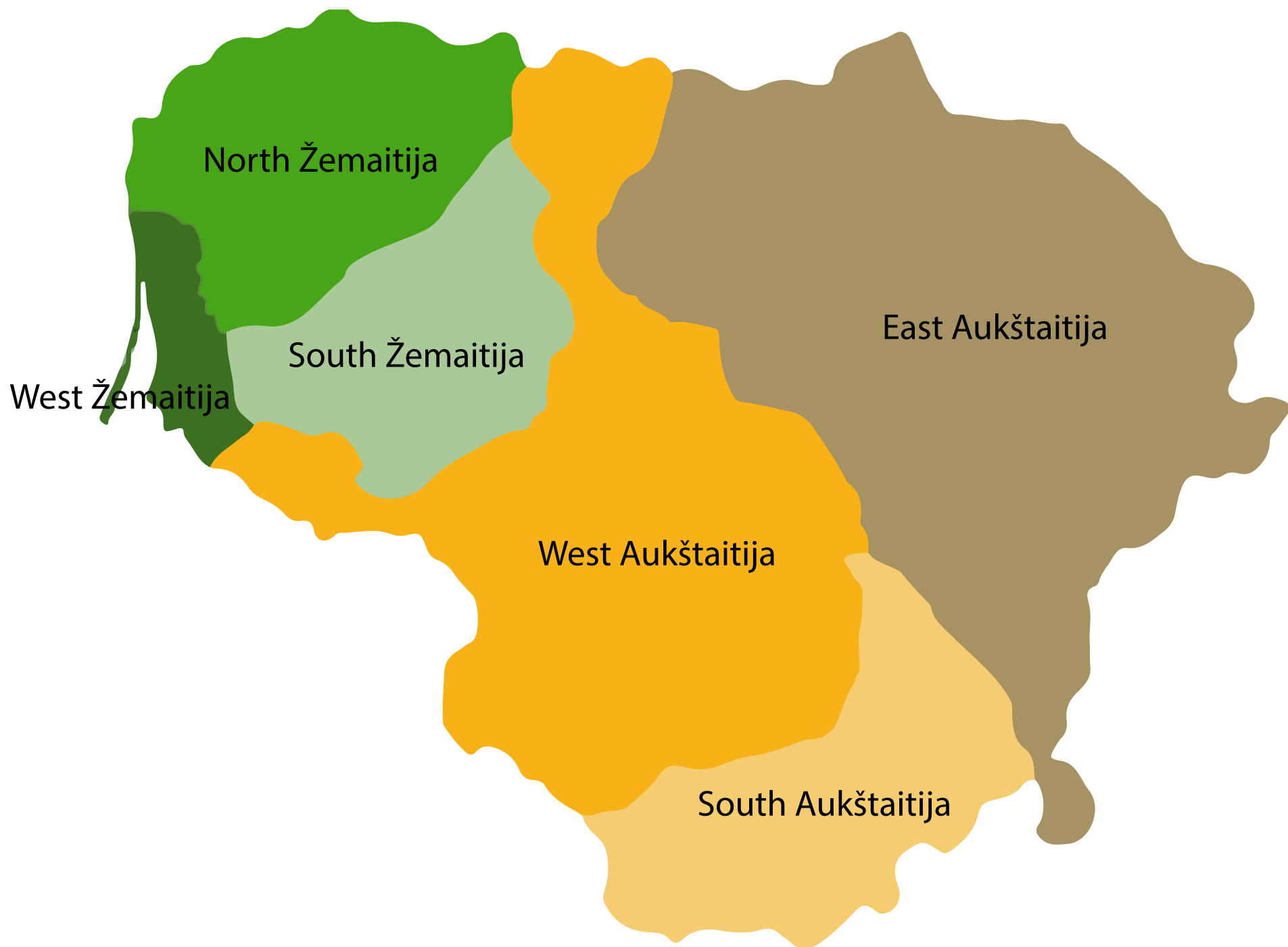

Supplement: Additional file 3: — Map of ethnolinguistic groups in the Lithuanian population. Six ethnolinguistic groups are distinguished in Lithuania: three groups of Aukštaičiai (East, South, West) and three groups of Žemaičiai (North, South, West). (PDF 317 kb) [file 12863_2016_373_MOESM3_ESM.pdf]
